# Preeclampsia and Extracellular Vesicles

**DOI:** 10.1007/s11906-016-0678-x

**Published:** 2016-09-02

**Authors:** Sarwat I. Gilani, Tracey L. Weissgerber, Vesna D. Garovic, Muthuvel Jayachandran

**Affiliations:** 1 Department of Internal Medicine, Division of Nephrology and Hypertension, Mayo Clinic, Rochester, MN 55905 USA; 2 Department of Surgery, Mayo Clinic, 200 First Street SW, Rochester, MN 55905 USA; 3 Department of Physiology and Biomedical Engineering, Mayo Clinic, Rochester, MN 55905 USA

**Keywords:** Hypertensive pregnancy disorder, Microvesicles, Exosomes, Cell-cell communication, Vesicles

## Abstract

Preeclampsia is a hypertensive pregnancy disorder characterized by development of hypertension and proteinuria after 20 weeks of gestation that remains a leading cause of maternal and neonatal morbidity and mortality. While preeclampsia is believed to result from complex interactions between maternal and placental factors, the proximate pathophysiology of this syndrome remains elusive. Cell-to-cell communication is a critical signaling mechanism for feto-placental development in normal pregnancies. One mechanism of cellular communication relates to activated cell-derived sealed membrane vesicles called extracellular vesicles (EVs). The concentrations and contents of EVs in biological fluids depend upon their cells of origin and the stimuli which trigger their production. Research on EVs in preeclampsia has focused on EVs derived from the maternal vasculature (endothelium, vascular smooth muscle) and blood (erythrocytes, leukocytes, and platelets), as well as placental syncytiotrophoblasts. Changes in the concentrations and contents of these EVs may contribute to the pathophysiology of preeclampsia by accentuating the pro-inflammatory and pro-coagulatory states of pregnancy. This review focuses on possible interactions among placental- and maternal-derived EVs and their contents in the initiation and progression of the pathogenesis of preeclampsia. Understanding the contributions of EVs in the pathogenesis of preeclampsia may facilitate their use as diagnostic and prognostic biomarkers.

## Introduction

Preeclampsia is characterized by new-onset hypertension (systolic blood pressure ≥140 mmHg/diastolic blood pressure ≥90 mmHg), with either proteinuria (≥300 mg/24 h) and/or organ dysfunction after 20 weeks of gestation [1]. The underlying cellular and molecular mechanisms that trigger preeclampsia and facilitate its progression are not well understood. Consequently, there are no established early diagnostic tests or effective targeted pharmacological treatments for preeclampsia. The only treatment option is delivery. With a global prevalence rate of 2.7–8.2 % of pregnancies, preeclampsia remains a major challenge in patient management for physicians [2–4].

**Fig. 1:**
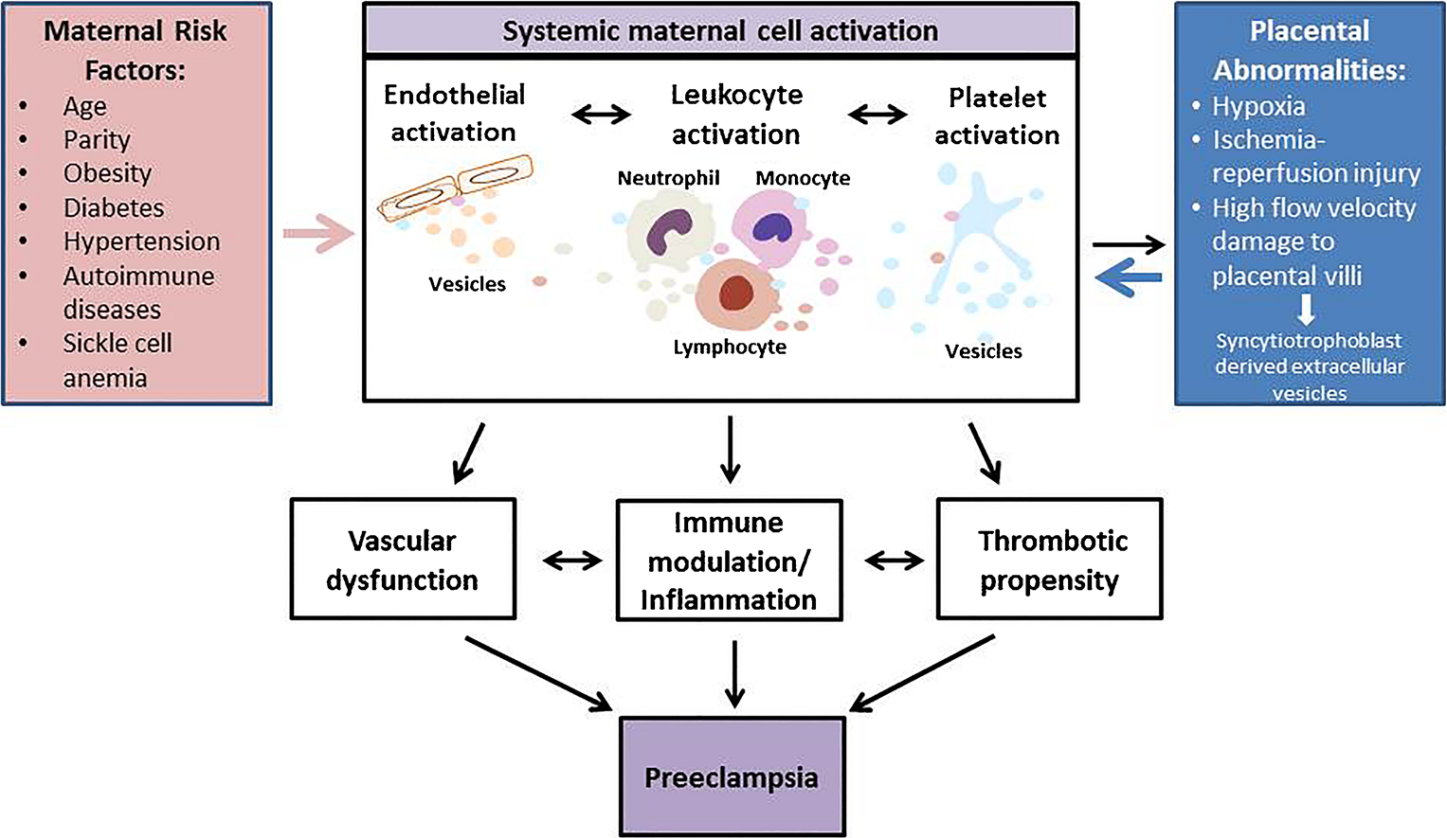
Role of EVs in pathogenesis of preeclampsia. Maternal risk factors and placental abnormalities cause systemic maternal cell activation resulting in release of EVs. Endothelial-, leukocyte-, and platelet-derived EVs give rise to vascular dysfunction, immune modulation, and increased thrombotic propensity. These processes collectively contribute to progression of pathogenesis of preeclampsia

It is recognized increasingly that preeclampsia is a heterogeneous disease, caused by several distinct underlying mechanisms that may result in different clinical phenotypes [5••]. This is reflected in current clinical practice, as it is common to divide preeclampsia into early (<34 weeks of gestation) and late (>34 weeks of gestation) preeclampsia based on the timing of the onset of symptoms. Similarly, preeclampsia may be classified as mild or severe depending on the severity of symptoms, including blood pressure (mild, <160/110 mmHg; severe, ≥160/110 mmHg), and the presence or absence of organ dysfunction (kidney failure, liver rupture, stroke, and seizure). Studies investigating the etiologies of preeclampsia have hypothesized that this syndrome may have placental and maternal forms [6]. This approach takes into account the underlying mechanisms. It has been proposed that defects in remodeling of the maternal spiral arteries that supply the placenta ultimately lead to placental ischemia [7••], ischemia reperfusion injury [8], or high velocity blood flow injury in the intervillous space [9••] in placental preeclampsia. This triggers the release of one or more placental factors that cause systemic endothelial dysfunction in the maternal circulation. Alternatively, maternal preeclampsia may arise in the setting of vascular dysfunction, oxidative stress, and metabolic abnormalities, such as hypertension, obesity, or diabetes that predate or are exacerbated by pregnancy (in the text that follows, we will refer to these conditions as preeclampsia risk factors). Endothelial dysfunction worsens with advancing gestation as the mother is unable to adapt to the physiological stress of pregnancy (Fig. 1). Placental preeclampsia is commonly viewed as early or severe, while maternal preeclampsia is sometimes characterized as late or mild. Although the dichotomous view of preeclampsia is overly simplistic, the relative contributions of maternal vs. placental factors likely differ among individual women, ultimately resulting in a diverse spectrum of clinical presentations. Irrespective of the predominant underlying mechanism, the interactions among maternal and placental pathophysiological factors may lead to a vicious cycle of maternal inflammation, vascular dysfunction, and the activation of pro-coagulation pathways that ultimately cause the symptoms and signs of preeclampsia.

Based on the complex nature of the origin of preeclampsia, we hypothesize that placental and maternal cells cross-talk, mediated by extracellular vesicles (EVs), contributes to the initiation and progression of preeclampsia in women, both with and without known pre-existing risk factors (Fig. 1). In women for whom EVs derived from the placenta are the major contributors, we propose that the symptoms of preeclampsia may appear earlier in gestation. If EVs derived from maternal cells are the major contributors, the symptoms may appear later in gestation. Two distinct types of EVs (exosomes and microvesicles) are released by almost all activated cells or cells involved in pathophysiological processes [10–13]. Exosomes and microvesicles differ in size and their modes of formation. Exosomes are smaller than microvesicles (30–120 nm vs. 40–1000 nm) [12], and are formed by the endocytosis of multivesicular bodies and are released from cells by exocytosis. In contrast, microvesicles (MVs) are membrane-bound vesicles that are shed from the plasma membrane [12]. Despite these differences, the size ranges for these two distinct classes of EVs overlap in some extent, and there are currently no established methods available to distinguish them purely on basis of size. Surface-specific EV markers that can be used to differentiate microvesicles and exosomes have not yet been identified. We have therefore used the term EVs, as previously suggested by the scientific community [14], to refer to exosomes and microvesicles in this review.

As the role of EVs in the pathophysiology of preeclampsia is an emerging field, the literature contains conflicting data. This review focuses on the most consistent findings, while providing an overview of areas with disparate findings.

## Maternal Cell-Derived EVs Before Pregnancy

Changes in circulating EVs offer a unique opportunity to examine how preeclampsia risk factors affect the functions of the parent cells and tissues prior to and during pregnancy. Risk factors for preeclampsia include obesity, pre-gestational diabetes mellitus, hypertension, and systemic lupus erythematosus (Table 1). These risk factors could alter the functioning of different types of maternal cells prior to pregnancy, as demonstrated by changes in the concentrations and bioactive molecular contents of the circulating EVs. Studies examining EVs in non-pregnant women suggest that risk factors for preeclampsia are associated with changes in EVs derived from vascular endothelial cells, leukocytes, and platelets [17–26] (Table 2). As shown in Table 2, compared to non-pregnant women without preeclampsia risk factors, non-pregnant women with these risk factors are reported to have increased endothelial-derived EVs [17–19, 22, 24, 25, 27]. Similarly, non-pregnant women with these risk factors are reported to have increased platelet-derived EVs [17, 20–22, 26]; however, they have either increased or decreased concentrations of leukocyte-derived EVs [20, 23–25].

**Table 1:** Preeclampsia-associated maternal risk factors

Pre-pregnancy maternal characteristics	Relative risk	Reference
Obesity	2.47	Duckitt et al. [15]
Pre-gestational diabetes mellitus	3.56	Duckitt et al. [15]
Hypertension	1.38–2.37	Duckitt et al. [15]
Autoimmune diseases		
• Systemic lupus erythematosus	–	
• Antiphospholipid syndrome	9.72	Duckitt et al. [15]
Sickle cell anemia	2.43	Oteng-Ntim et al. [16]
Nulliparity	2.91	Duckitt et al. [15]
Preeclampsia in prior pregnancy	7.19	Duckitt et al. [15]
Family history of preeclampsia	2.90	Duckitt et al. [15]

**Table 2:** EVs in non-pregnant women with preeclampsia-associated risk factors compared to non-pregnant women without preeclampsia-associated risk factors

Risk factor	Extracellular vesicles	Results	Reference
Obesity	Endothelial-derived	↑	Stephanian et al. [17]
	Platelet-derived	↑	Stephanian et al. [17]
Diabetes mellitus	Endothelium-derived	↑	Sabatier et al. [18]
		↑	Tramontano et al. [19]
	Leukocyte-derived	No difference	Zhang et al. [20]
	Platelet-derived	↑	Zhang et al. [20]
		↑	Strano et al. [21]
Hypertension	Endothelium-derived	↑	Preston et al. [22]
	Platelet-derived	↑	Preston et al. [22]
Systemic lupus erythematosus	Leukocyte-derived	↑	Lacroix et al. [27]
		↓	Neilson et al. [24]
	Endothelium-derived	↑	Lacroix et al. [27]
		No difference	Neilson et al. [24]
	Platelet-derived	↑	Stephanian et al. [17]
Antiphospholipid syndrome	Endothelium-derived	↑	Dignat-George et al. [25]
	Leukocyte-derived	↑	Dignat-George et al. [25]
Sickle cell anemia	Platelet-derived	↑	Wun et al. [26]

Studies have shown that the effects of preeclampsia-associated risk factors are similar to the effects of preeclampsia on endothelial-derived EVs. Women with both preeclampsia-associated risk factors and preeclampsia are reported to have increased concentrations of endothelial-derived EVs when compared to either non-pregnant women without these risk factors and/or normotensive pregnant women, respectively [17–19, 22, 24, 25, 27–29]. However, some studies report no change in endothelial-derived EV concentrations in women with preeclampsia compared to normotensive pregnant women [30–32]. Concentrations of leukocyte-derived EV (LEV) in non-pregnant women with preeclampsia-associated risk factors are reported to be increased, decreased, or not changed, when compared to non-pregnant women without these risk factors [20, 23–25]. Whereas in women with preeclampsia, LEV concentrations are reported to be increased compared to normotensive pregnant women [28, 33]. The effects of preeclampsia-associated risk factors are reported to be opposite to the effects of preeclampsia on platelet-derived EV (PEV). Increased concentrations of PEV are present in women with preeclampsia-associated maternal risk factors compared to non-pregnant women without these risk factors. However, PEV concentrations are reported to be decreased in women with preeclampsia compared to normotensive pregnant women [17, 20–22, 26].

Use of low-dose aspirin is recommended for women with preeclampsia-associated risk factors to decrease the morbidity and mortality associated with preeclampsia [34]. The beneficial effects of aspirin in those with preeclampsia-associated risk factors may, at least in part, be explained by the effect of aspirin on platelet activity. By inhibiting thromboxane A_2_ synthesis, aspirin decreases platelet activation and, in turn, likely affects the production of PEV. Understanding the effect of aspirin on PEV production and content in women with preeclampsia-associated risk factors may delineate the mechanistic pathways by which PEV contribute to the pathogenesis of preeclampsia.

## Placenta-Derived EVs

The placenta plays a critical role in the pathophysiology of preeclampsia [35]. Placental trophoblasts are involved in spiral artery remodeling and differentiate into extravillous trophoblasts and villous trophoblasts. The villous trophoblasts fuse to form syncytiotrophoblasts. The extravillous trophoblasts invade the distal portions of the spiral arteries, displacing maternal vascular endothelial and smooth muscle cells [36, 37]. This process transforms the distal portions of the spiral arteries from narrow vessels into wide, flaccid conduits [38, 39•]. The uterine oxygen gradient in early pregnancy favors extravillous trophoblast invasion of the uterine spiral arteries and spiral artery remodeling [40]. It is speculated that placental trophoblast-derived EVs (40–300 nm) [41] may also have a role in spiral artery remodeling [42]. Salomon et al. have shown that oxygen tension regulates the number and protein content of exosomes released by the placenta, with greater release of exosomes by placental trophoblasts under hypoxic conditions in vitro [43, 44]. Placental exosomes are reported to contain serine proteases and metalloproteases (MMP), including MMP-12 [44]. It has been hypothesized that MMP-12 secreted by trophoblasts may facilitate trophoblast invasion by contributing to the remodeling of the extracellular matrix in the vascular wall [45].

Abnormal placentation in women with preeclampsia may increase circulating concentrations of placental-derived EVs. Studies have shown higher concentrations of syncytiotrophoblast- derived EVs [28, 46, 47•, 48], with altered lipid and protein content [49, 50], in women with early-onset or severe preeclampsia compared to normotensive pregnant women. In addition, studies suggest that the syncytiotrophoblast apoptosis rate is elevated in preeclampsia (5–6 %) when compared to normal pregnancy (2–3 %) [51]. In accordance with this finding, higher circulating concentrations of syncytiotrophoblast-derived EVs have been reported in preeclamptic women compared to normotensive pregnant women [41, 46, 47•]. Furthermore, women with early-onset preeclampsia seem to have higher syncytiotrophoblast-derived EVs concentrations than women with late-onset preeclampsia [28, 48, 52•]. Further studies are needed to identify the exact cellular or molecular pathways that stimulate production of placental-derived EVs, which may contribute to the development of preeclampsia.

## Effect of Maternal EVs on Production of Placental-Derived EVs

Dynamic interactions between maternal and fetal factors are constantly occurring at the maternal-fetal interface. These interactions contribute to the regulation of trophoblast phenotype and endovascular invasion. Chemokines and their receptors (CXCR4, CXCR7, CXCL12) promote cell survival and proliferation and inhibit apoptosis [53]. Lu et al. [53] have shown decreases in the expressions of CXCR4, CXCR7, and CXCL12 molecules in trophoblast cells obtained from the placentas of preeclamptic women. The causes of these decreases are not known. One possible mechanism may be that circulating maternal factors down-regulate chemokine receptors at the post-transcriptional level. Alternatively, downregulation of molecules or upregulation of molecular inhibitors at the transcriptional level can also occur in trophoblasts, as demonstrated by Zhou et al. [54]. This study also observed an upregulation of the angiogenesis inhibitor SEMA3B in trophoblasts obtained from women with preeclampsia. SEMA3B inhibits trophoblast invasion of vessels by promoting trophoblast apoptosis. It additionally showed that the increased levels of SEMA3B from preeclamptic trophoblasts returned to control levels after 48 h in a culture system. Based on this finding, the authors proposed that factors in the maternal milieu cause reversible upregulation of SEMA3B in trophoblasts in preeclampsia [54]. The role of maternal cell-derived EVs in regulating trophoblast gene expression in preeclampsia remains to be determined. Holder et al. [55] have shown that placental trophoblasts take up exosomes from maternal macrophages and alter the placental production of inflammatory cytokines. Given this evidence, we hypothesize that EVs derived from maternal cells have the potential to alter trophoblast gene expression and function. These changes may contribute to defective trophoblast invasion and increased trophoblast apoptosis.

Placental debris is cleared by macrophages at the maternal-fetal interface [56]. It is known that syncytiotrophoblast-derived vesicles affect the functions of maternal cells, including platelets, leukocytes, erythrocytes, and endothelial cells [57•, 58–62]. Therefore, it is possible that EVs produced by syncytiotrophoblasts also contribute to regulating maternal macrophage activity at the maternal-fetal interface. Exploring the effects of syncytiotrophoblast-derived EVs on maternal macrophages will elucidate the mechanisms by which macrophage activity is regulated at the maternal-fetal interface. The role of maternal EVs in the regulation of trophoblast turnover and immune activity at the maternal-fetal interface will provide valuable insight into the factors regulating the dynamics of the maternal-fetal interface. This, in turn, will delineate the role that EVs of maternal and placental origins and their interactions have in the initiation and progression of placental preeclampsia.

## Maternal Cell-Derived EVs and Their Interactions

### Platelet-Derived EVs

Platelets are the largest source of EVs in blood in healthy non-pregnant women [63]. When compared to non-pregnant women, most studies report lower platelet-derived EV(PEV) concentrations in normotensive pregnant women [28, 64]. While most studies have observed further reductions in PEV in women with preeclampsia [28, 31, 32, 65], a few studies have shown higher concentrations of PEV, or no change [66, 67] (Table 3). It is plausible that differences in the reported concentrations of PEV among the studies are reflective of different preeclampsia subtypes and their underlying mechanisms. Three different theories have been proposed to explain the lower concentrations of PEV with preeclampsia. (1) Lower platelet counts in preeclampsia may contribute to lower concentrations of PEV [65]. (2) Some studies have hypothesized that the lower concentrations of PEV may partly be due to increased trapping or participation of PEVs in thrombin generation and fibrin clot formation [28, 64, 67, 72]. (3) It is postulated that lower PEVs may be due to their association or binding with leukocytes [65].

**Table 3:** Comparison of EVs between preeclampsia and normotensive pregnancy

Parameter	Preeclampsia	Normotensive pregnancy	Reference
Total EVs	Increased/no change/decreased	Present	Tesse et al. [68]; Marques et al. [28]; Mikhailova et al. [33]; VanWijk et al. [69]; Holthe et al. [70]; Bretelle et al. [32]; Lok et al. [31]
Syncytiotrophoblast-derived EVs (early-onset PE)	Increased/no significant change	Present	Knight et al. [46]; Germain et al. [47•]; Goswami et al. [48]; Lok et al. [31]
Syncytiotrophoblast-derived EVs (late-onset PE)/severe	No change/no change	Present	Goswami et al. [48]; [28]
Endothelial cell-derived EVs	Increased/no change	Present	Marques et al. [28]; Gonzalez et al. [29]; VanWijk et al. [69]; Bretelle et al. [32]; Lok et al. [31]
Platelet-derived EVs	Decreased	Present	Marques et al. [28]; Bretelle et al.[32]; Lok et al.[28, 31]; Lok et al. [65]
Leukocyte-derived EVs	Increased	Present	Mikhailova et al. [33]; Marques et al. [28]
• Granulocyte-derived EVs	Increased	Present	Lok et al. [71]; vanWijk et al. [69]; Marques et al. [28]
• Monocyte-derived EVs	Increased	Present	Lok et al. [31, 71]; Marques et al.[28]
• Lymphocyte-derived EVs	Decreased	Present	Lok et al. [71]; Marques et al.[28]
• T cell-derived EVs	Increased	Negligible	vanWijk et al.[69]
• T helper cell-derived EVs	Increased	Negligible	Lok et al. [31]
Erythrocyte-derived EVs	Increased	Present	Lok et al. [31]; Marques et al. [28]; Dragovic et al. [64]

### Leukocyte-Derived EV

Studies have shown that leukocyte counts and concentrations of leukocyte-derived EV (LEV) are higher in normotensive pregnant women compared to non-pregnant women [28, 33, 64, 69, 71, 73]. The inflammatory state of normotensive pregnancy is further exacerbated in preeclampsia, as preeclamptic women have even higher leukocyte counts and concentrations of LEV [64, 71] (Table 3). The upregulation of granulocytes (neutrophils)-and monocyte- and granulocyte-derived EVs-has been suggested [28, 33, 71] to aid in the removal and regulation of syncytiotrophoblast-derived vesicles and placental debris that are released into the maternal circulation. Pro-inflammatory EVs can be produced by endothelial cells in response to inflammatory stimuli [74]. Therefore, we speculate that the pro-inflammatory state in preeclampsia may increase circulating concentrations of endothelial-derived pro-inflammatory EVs. These pro-inflammatory EVs may contribute to the increases in the peripheral blood neutrophils and monocytes in preeclampsia, facilitating the immunomodulation and upregulation of phagocytosis.

The role of EVs in leukocyte activation has also been explored. Studies have shown that syncytiotrophoblast-derived vesicles from normal pregnancy and preeclampsia cause leukocyte activation in vitro [47•, 75] that is mediated by toll-like receptors and nuclear factor (NF-Kβ) [76]. Peripheral blood EVs have also been shown to alter monocyte phenotype in vitro [60]. Furthermore, it has been shown that activated leukocytes produce inflammatory cytokines (IL-1, IL-8) and nuclear factor (NF-Kβ) that can stimulate EV production [77]. The circulating EVs of maternal and placental origins most likely propagate inflammation in preeclampsia [37].

### Red Blood Cells (Erythrocytes)-Derived EV

While most studies show an increase in the concentrations of red blood cells (erythrocytes)-derived EV (REV) in normotensive pregnant women and preeclamptic women compared to non-pregnant women [28, 31, 64], one study showed no difference [64]. Increased REV concentrations in pregnancy suggest erythrocyte activation, which may be due to increased oxygen demand in pregnancy or stimulation by circulating EVs. Alternatively, increased REV concentrations in preeclampsia may be due to erythrocyte disruption and hemolysis [78], which may be associated with widespread thrombosis [28]. Ten to twenty percent of women with preeclampsia develop hemolysis, elevated liver enzymes, and low platelets (HELLP) syndrome, which is characterized by erythrocyte disruption [79]. Hemolysis, in addition, may result from an autoimmune reaction to trophoblast-derived vesicles deposition on erythrocytes that may explain the increased concentrations of REV in preeclampsia. Determination of REV concentrations and composition in pregnant women with and without preeclampsia and with HELLP syndrome may help to elucidate the mechanisms by which preeclampsia progresses to HELLP syndrome.

### Vascular Endothelium and Smooth Muscle Cell-Derived EVs

The concentrations of endothelial-derived EV (EEV) are lower in normotensive pregnant women compared to non-pregnant women, reflecting either decreased production of EEV in normotensive pregnancy or EEV binding to circulating blood cells (platelets and leukocytes). Decreased EEV production is associated with decreased peripheral vascular resistance in normotensive pregnancy [80, 81]. Estrogen and maternal fluid dynamics [82] have been postulated to decrease EEV production in normal pregnancy. As syncytiotrophoblast-derived vesicles carry bioactive molecules (e.g., mRNA or miRNA), it may be possible that they regulate the endothelial functions associated with decreased peripheral vascular resistance. Petrozella et al. [83] and VanWijk et al. [69] have shown that circulating EEV concentrations were higher in preeclamptic women compared to normotensive pregnant women (Table 3). This indicates activation of endothelial cells in preeclampsia and an association between increased concentrations of EEV and vascular dysfunction. It has been demonstrated that EEV have differing characteristics depending on signaling stimulus and associated thrombotic and inflammatory processes or conditions [84]. In addition to negatively charged phospholipids, the surface of EEV display receptors (E-selectin, intercellular adhesion molecule-1, and vascular cellular adhesion molecule-1) and markers expressed by endothelial cells. Determining the composition and surface expressions of EEV in normotensive pregnant and preeclamptic women may elucidate the interactions that result in the exacerbation of inflammation and coagulation activation in preeclampsia. EEVs are implicated in the progression of inflammatory vascular diseases [85]. EEV-mediated communication between endothelial cells and the target cells (leukocytes, platelets) is vital to the understanding of the exaggerated pro-inflammatory and pro-coagulation states underlying preeclampsia. Vascular smooth muscle cell-derived EVs (SMCEVs) have also been implicated in pathological processes resulting in vascular disease progression [86]. The contributions of SMCEVs in the pathophysiology of preeclampsia have not been investigated. Studies exploring the role of SMCEVs may delineate additional mechanistic vascular pathways contributing to the initiation and progression of preeclampsia.

## EVs and Coagulation in Preeclampsia

The pro-coagulation state of normotensive pregnancy is associated with a decrease in fibrinolytic activity caused by increased pro-coagulant factors and fibrinolytic inhibitors (e.g., plasminogen activator inhibitor-1, [PAI-1]). In addition, the phosphatidylserine present on the surfaces of placental and maternal cell-derived EVs contributes to the hypercoagulable state. Thrombin generation and prothrombin fragments increase in normotensive pregnancy [87]. This hypercoagulable state is exaggerated in preeclampsia, resulting in widespread blood clot formation [88], with fibrin deposition in the maternal vasculature, organs, and the placenta [89]. This may be due to the increased total concentrations of pro-coagulant surface positive EVs; however, certain studies have shown no change or even decreases in total counts of MVs in preeclampsia. Alternatively, changes in the phenotype of EVs could also cause increased coagulation. Certain pro-coagulatory molecules (e.g., PAI-1) also have important roles in EEV generation [90, 91]. Studies exploring the link between pro-coagulant factors and EV generation in preeclampsia may elucidate the mechanism that links endothelial dysfunction with widespread coagulation. Depending upon the severity of preeclampsia, widespread coagulation activation and clot formation results in ischemic damage in end organs, as well as widespread disseminated intravascular coagulation.

Tissue factor is a ubiquitous 47 kDa transmembrane protein that initiates the inflammatory coagulation pathway. It is present on cells, as well as on cell-derived EVs. It is constitutively expressed in some cells (perivascular fibroblasts) and conditionally expressed in other cells in response to a variety of stimuli, including activated monocytes, macrophages, and the vascular endothelium [92]. The EVs released from activated leukocytes and endothelial cells also express tissue factor [63]. Previous studies have demonstrated that tissue factor is present on the surface of syncytiotrophoblast-derived vesicles [93, 94]. Upregulation of tissue factor on syncytiotrophoblasts occurs in preeclampsia [95], which is associated with the increased activity of tissue factor in preeclampsia [96]. Gardiner et al. [96] have demonstrated higher tissue factor activity and thrombin generation associated with syncytiotrophoblast-derived vesicles from preeclamptic women compared to normotensive pregnant women. Preclinical studies have revealed improved clinical outcomes following anticoagulant therapies in animal models of preeclampsia [97]. Human studies that explore the use of anticoagulants suitable for preeclamptic women are needed. Based on animal studies, anticoagulant therapy has the potential to improve maternal and fetal outcomes in preeclamptic pregnancies.

## Conclusions

EVs have a dynamic role in the communication among maternal vascular cells (the vascular endothelium, circulating leukocytes, and platelets) and the placenta, thus contributing to the progression of normal pregnancy. Depending on pre-existing maternal conditions, any of these vascular components during pregnancy may be capable of initiating the cascade of events that result in preeclampsia. In maternal conditions associated with the activation of vascular endothelial cells and immune system modulation, EEVs can augment inflammation, coagulation, and endothelial dysfunction. Pre-pregnancy maternal platelet activation can augment endothelial dysfunction and inflammation via PEVs, facilitating the progression to preeclampsia. In women without maternal risk factors associated with preeclampsia, it is possible that placental trophoblast-derived EVs may contribute to the maternal milieu that favors progression to preeclampsia.

### Future Directions

The complex interactions of maternal cell-derived EVs and placental-derived EVs need to be explored further to elucidate the mechanisms of initiation and progression of preeclampsia, with and without known maternal risk factors. In addition to quantitative alterations in EVs, characterizing the bioactive molecular contents (mRNA, miRNA, proteins, lipids, and metabolites) of these EVs based on their cellular origins and their interactions with target cells in the blood and vascular compartments may help to identify underlying mechanisms that contribute to the pathophysiology of preeclampsia. Furthermore, understanding the roles of the specific types of EVs in the pathogenesis of preeclampsia may enable the development of a panel of biomarkers that will help to identify pregnant women at risk for developing preeclampsia. In addition, as maternal vascular, immune, and coagulation systems may have EV-mediated bidirectional communication, it may be possible to therapeutically target one component of the maternal system, which may facilitate the other two systems to return to normalcy in preeclampsia. As most EVs in the healthy pregnant state are derived from platelets, therapeutic interventions aimed at stabilizing platelets can correct the exaggerated pro-inflammatory and hypercoagulable state in preeclampsia that may also help to mitigate the vascular dysfunction and immune flare.
